# Multivariable Coupled System Control Method Based on Deep Reinforcement Learning

**DOI:** 10.3390/s23218679

**Published:** 2023-10-24

**Authors:** Jin Xu, Han Li, Qingxin Zhang

**Affiliations:** School of Artificial Intelligence, Shenyang Aerospace University, Shenyang 110136, China; sh_xujin@126.com (J.X.); 15840222126@163.com (H.L.)

**Keywords:** multivariate coupled system, deep reinforcement learning, control system, PPO, normalization

## Abstract

Due to the multi-loop coupling characteristics of multivariable systems, it is difficult for traditional control methods to achieve precise control effects. Therefore, this paper proposes a control method based on deep reinforcement learning to achieve stable and accurate control of multivariable coupling systems. Based on the proximal policy optimization algorithm (PPO), this method selects tanh as the activation function and normalizes the advantage function. At the same time, based on the characteristics of the multivariable coupling system, the reward function and controller are redesigned structures, achieving stable and precise control of the controlled system. In addition, this study used the amplitude of the control quantity output by the controller as an indicator to evaluate the controller’s performance. Finally, simulation verification was conducted in MATLAB/Simulink. The experimental results show that compared with decentralized control, decoupled control and traditional PPO control, the method proposed in this article achieves better control effects.

## 1. Introduction

For single-input single-output (SISO) systems, conventional controllers are widely employed in process control. This control methodology is founded on fundamental principles, namely that it is facile to design and expedient to debug. It has found extensive application in industrial process control and has achieved significant success [1]. However, with the development of industry, the scale of production continues to expand, and the complexity of systems increases. Most of these systems are multi-input multi-output (MIMO) systems, involving multiple manipulated variables (MVs) and controlled variables (CVs) that are interconnected. Due to the complexity of the system’s structure, it is difficult to obtain precise mathematical models, and the controlled processes often exhibit some degree of nonlinearity. Therefore, when controlling the system by dividing it into several SISO systems, the internal correlations of the system, model uncertainties, and partial nonlinearity are often overlooked. Although this drawback can be overcome by feedback control, for certain systems, the characteristics of these multivariable systems are pronounced, and using only SISO control methods may not yield satisfactory results. Therefore, researching control methods for MIMO systems and applying them in industrial process control is crucial for improving production efficiency and ensuring safe and reliable operation.

Currently, there are several control methods for MIMO systems, including decentralized control [2,3], decoupling control [4,5,6], robust control [7,8,9], model predictive control (MPC) [10,11], and active disturbance rejection control (ADRC) [12,13]. For decentralized control methods, the controller is in a diagonal form and does not necessitate the addition of a decoupling compensator. This method is commonly employed in industrial processes. In contrast to tuning parameters for a single-loop PID controller, parameter tuning for a decentralized PID controller must consider loop coupling [14], which increases the complexity of parameter tuning. The decentralized control structure has advantages such as fewer control parameters, simplicity in terms of structure, and easy implementation. Therefore, in cases where the degree of system coupling is low, the decentralized control structure has been widely applied. The decoupling control method involves adding a decoupling controller between the decentralized control structure and the controlled object, which decouples the controlled object into a diagonal or diagonally dominant form. As a result, the decoupled system can be designed with PID parameters for each individual loop, like the SISO method. However, due to uncertainties in the actual control process, it is not possible to construct a completely dynamic decoupler with a decoupling compensator. Even if an appropriate form of the decoupling controller can be obtained, its structure becomes complex, making it difficult to implement in practice. Robust control is a control method that addresses system uncertainties and external disturbances, aiming to maintain system stability and performance. It can provide good control performance in the presence of parameter uncertainties, external disturbances, or measurement noise [15]. However, robust control design is relatively complex as it requires consideration of the uncertainty models and ranges of the system. In controller design, robustness analysis and appropriate selection of stability conditions need to be performed. MPC is a control method based on predictive models that can handle multivariable systems and constraints. It optimizes control input sequences to minimize predetermined performance metrics within a prediction time window [16]. However, MPC involves high computational complexity as it requires the real-time solving of optimization problems. Additionally, implementing MPC control necessitates accurate system models and measurement data, which can be challenging to obtain for complex systems. ADRC is a control method that suppresses the impact of disturbances on the system. It achieves accurate control by estimating and compensating for various uncertainties and disturbances present in the system. ADRC methods usually do not rely on detailed mathematical models of the system, making them robust and applicable. However, the design of ADRC controllers is relatively complex, and they may not be as sensitive to high-frequency disturbance responses of the system. As the complexity of the controlled objects increases, high-dimensional, high-order multivariable systems may exist, and most systems exhibit time delays in each loop. The design of controller structures for such systems becomes even more complex. Therefore, it is necessary to research new control strategies.

In recent years, with the continuous development of artificial intelligence technology, reinforcement learning (RL), as a trial-and-error-based machine learning approach, has brought new opportunities for controlling multivariable coupled systems due to its powerful nonlinear modelling and adaptive learning capabilities [17]. For example, Yang et al. [18] addressed the multivariable tracking control problem in wastewater treatment processes using reinforcement learning control based on direct heuristic dynamic programming (DHDP). This method uses heuristic information to guide the search process of dynamic programming. In traditional dynamic programming, it is usually necessary to traverse the entire state space, which can be very expensive for large-scale problems. DHDP accelerates the search by selectively exploring a portion of the state space chosen based on heuristic information, making it more efficient in finding policies. However, DHDP’s performance is highly dependent on the quality of the heuristic function. It tends to search the state space guided by heuristic information, which can lead to the algorithm getting stuck in local optima and failing to find the global optimal policy. Thayumanavan et al. [19] developed a general data-driven adaptive PID controller by combining reinforcement learning with PID controllers, where PID parameters are learned and adjusted to control the system. However, in industrial settings, most systems are complex, multivariable, coupled, and laggy, and this method may result in imprecise and unstable system control. Zhu et al. [20] implemented intelligent, direct thrust control for multivariable turbofan engines using proximal policy optimization (PPO), a deep reinforcement learning algorithm. However, this method did not deeply investigate the impact of the PPO algorithm’s activation and advantage functions on system performance. Therefore, this paper proposes a deep-reinforcement-learning-based control method for multivariable coupled systems. The method employs the PPO algorithm as the deep reinforcement learning controller and thoroughly investigates the influence of activation and advantage functions on system performance. This approach addresses the issues of imprecise and unstable system control caused by the coupling among variables in multivariable systems. The main contributions of this study are as follows:

(1) End-to-end control of multivariable coupled systems has been achieved by designing a control strategy based on deep reinforcement learning while considering the presence of external disturbance signals in natural systems, enhancing the system’s robustness against disturbances.
(2) The advantages of using the tanh activation function and normalizing the advantage function have been validated.
(3) The impact of the control signal amplitude output by different controllers on the actuator and the entire system in industrial processes has been thoroughly considered.
(4) The design of the deep reinforcement learning controller in this study does not require a model or specialized knowledge of industrial processes, making this control structure readily transferable and applicable as a standard.

The remaining sections of this paper are organized as follows: Section 2 introduces the fundamental knowledge of multivariable systems and analyzes the coupling characteristics of multivariable systems in detail through an example. Section 3 elaborates on the design details of the deep reinforcement learning controller. Section 4 discusses the effectiveness of the proposed control method. Section 5 provides a summary of this research and outlines future work.

## 2. Multivariate Coupled System

Over the past decades, the successful application of single-variable control theory has demonstrated the convenience and effectiveness of using transfer functions to express and analyze control systems. Therefore, in this study, transfer function matrices were employed as the tools to describe and analyze multivariable systems.

**Figure 1:**
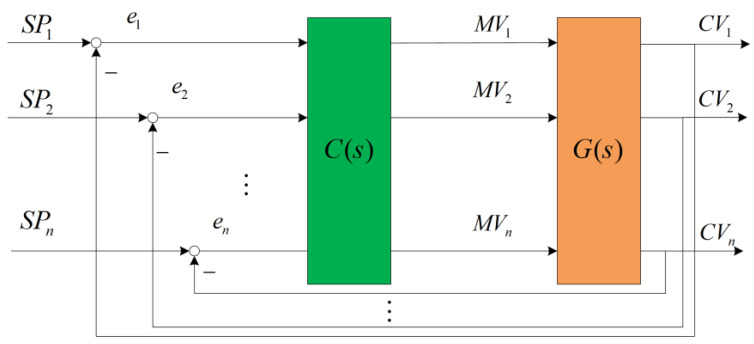
Block diagram of MIMO control system structure where C(s) represents the controller, and G(s) represents the plant (controlled object). Taking loop 1 as an example, $SP_{1}$ denotes the setpoint, $e_{1}$ represents the error between the setpoint $SP_{1}$ and the feedback controlled variable $CV_{1}$, and $MV_{1}$ represents the manipulated variable applied to the plant.

In a MIMO system, when a CV is only influenced by MV within its own loop and is independent of the MVs from other loops, meaning that the MV only affects the CV of their respective loop without affecting the CVs of other loops, then the system is considered to be decoupled. On the other hand, when there is mutual influence between systems, these systems are referred to as coupled systems. The block diagram of a MIMO system is shown in Figure 1.

If the plant G(s) is an uncoupled system, its transfer function matrix is given by:

(1) $$G(s)=\left[\begin{array}{cccc} G_{11}(s) & 0 & \cdots & 0\\ 0 & G_{22}(s) & \cdots & 0\\ & & \ddots & \\ 0 & 0 & \cdots & G_{nn}(s) \end{array}\right]$$

On the contrary, if the plant G(s) is a coupling system, its transfer function matrix is given by:

(2) $$G(s)=\left[\begin{array}{cccc} G_{11}(s) & G_{12}(s) & \cdots & G_{1n}(s)\\ G_{21}(s) & G_{22}(s) & \cdots & G_{2n}(s)\\ \vdots & & \ddots & \vdots \\ G_{n1}(s) & G_{n2}(s) & \cdots & G_{nn}(s) \end{array}\right]$$

where $G_{12}(s)$ represents the influence of $MV_{2}$ on $CV_{1}$, $G_{32}(s)$ represents the influence of $MV_{2}$ on $CV_{3}$, and so on.

**Figure 2:**
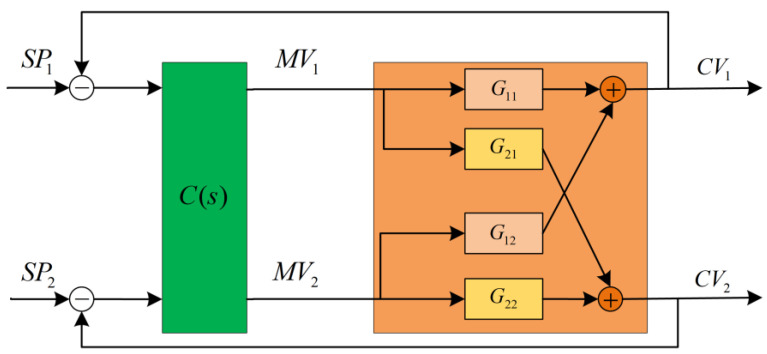
Two-input two-output system.

Taking a two-input, two-output (TITO) system as an example, the plant can be described by the transfer function matrix block diagram, as shown in Figure 2.

In the chemical reaction process of a certain reactor, the quantity of pure raw materials and the water content are two factors that affect the quick-drying property and strength of the concrete. In this case, the input control variables are represented as $MV_{1}$ for the quantity of pure raw materials and $MV_{2}$ for the water content, while the output variables are represented as $CV_{1}$ for the quick-drying property of the concrete and $CV_{2}$ for the strength of the concrete. The transfer function model [21] between the input and output variables of this multivariable system is given by:

(3) $$G(s)=\left[\begin{array}{ll} \frac{11}{7s+1}e^{-0.2s} & \frac{0.5}{3s+1}e^{-0.4s}\\ \frac{-3}{11s+1}e^{-0.2s} & \frac{0.3}{5s+1}e^{-0.4s} \end{array}\right]$$

where the static gain matrix of the system is:

(4) $$K=\left[\begin{array}{ll} G_{11}(s{)|}_{s\to 0} & G_{12}(s{)|}_{s\to 0}\\ G_{21}(s{)|}_{s\to 0} & G_{22}(s{)|}_{s\to 0} \end{array}\right]=\left[\begin{array}{ll} 11 & 0.5\\ -3 & 0.3 \end{array}\right]$$

The relative gain array Λ (RGA) of the system can be obtained from the static gain matrix. In automatic control systems, the relative gain array is a tool used to describe the relationships between various inputs and outputs in a control system.

(5) $$\Lambda =K\times {\left(K^{-1}\right)}^{T}=\left[\begin{array}{ll} 0.69 & 0.31\\ 0.31 & 0.69 \end{array}\right]$$

The selected variable pairings in the system are correct because the values on the main diagonal are closer to 1 compared to the positive values on the off-diagonal. Generally, when the relative gains are between 0.8 and 1.2, the coupling between the systems is considered to be weak. This coupling can be ignored in such cases, treating the multivariable system as multiple single-variable systems for control system analysis and design. However, the relative gains in this system are less than 0.8, indicating a strong coupling between the systems.

## 3. Controller Design Based on Deep Reinforcement Learning

### 3.1. Deep Reinforcement Learning

The core idea of reinforcement learning is for the agent to learn through trial and error to maximize long-term cumulative reward signals. Its advantage lies in automatically learning and adjusting strategies without human intervention. At a fundamental algorithmic level, the reinforcement learning can be divided into value-based and policy-based learning methods. Value-based or Q-learning methods are often used in discrete action spaces. They typically employ Monte Carlo and temporal difference estimations to learn value functions iteratively. On the other hand, value-distribution-based methods do not explicitly learn a policy. Instead, the policy can be directly defined as the action that maximizes the value function. Policy-based learning methods are more commonly used in continuous action spaces. Since the action space is continuous, traversing it to select the action that maximizes the Q-value is generally impossible. To address this problem, deep learning has been introduced into the reinforcement learning framework, giving rise to deep reinforcement learning (DRL) [22].

The reinforcement learning agent refers to the designed controller in control system terminology. The environment encompasses the system outside the controller, which refers to the multivariable coupled system. The controller represents the optimal control action sought by the designer. The design of the controller based on deep reinforcement learning relies on the design of the state, action, reward function, and the choice of deep reinforcement learning algorithm.

### 3.2. State

The state $s_{t}$ reflects essential information during the interaction between the agent and the environment. The selection of the state space directly affects the quality of the actions taken by the agent, thereby influencing the overall control effectiveness of the system.

Due to the presence of multiple loops in a multivariable coupling system, it is necessary to select appropriate state information for each loop to assist the agent in better learning the system characteristics. At a given time t, for loop $y_{1}$, the selected states $s_{t}$ include: the current error value $e_{t}^{1}$, the integral of the error at the current time $\int e_{t}^{1}dt$, the current actual value of the controlled variable $CV_{t}^{1}$, the current setpoint $SP_{t}^{1}$, and so on. For the TITO system in Figure 2, the state space S is defined as follows:

(6) $$S={\left[e_{t}^{1},\int e_{t}^{1}dt,CV_{t}^{1},SP_{t}^{1},e_{t}^{2},\int e_{t}^{2}dt,CV_{t}^{2},SP_{t}^{2}\right]}^{T}$$

### 3.3. Action

Action $a_{t}$ refers to the action taken by an agent in a specific state, and the primary objective of the agent is to select appropriate actions in diverse states with the aim of maximizing its long-term reward.

In this paper, action refers to the MVs in a multivariable coupling system. At a given time t, the action taken for loop $y_{1}$ is denoted as $MV_{t}^{1}$. For each loop, there is a corresponding action as an input. Therefore, for the TITO system in Figure 2, the action space is defined as follows:

(7) $$A={\left[MV_{t}^{1},MV_{t}^{2}\right]}^{T}$$

In most industrial process control systems, the action values are achieved through instruments and devices such as electric control valves or variable frequency pumps. If the amplitude of the action values is large, it can cause damage to the instruments and devices. Therefore, the magnitude of MV variation is also considered to be a criterion for evaluating the performance of a controller.

### 3.4. Reward

In reinforcement learning, the design of the reward function R is crucial as it defines the goals and feedback mechanism for the agent during the learning process. It guides the agent on which actions to take in the environment and how to adjust its learning based on the feedback received. Thus, it influences the agent’s future decision-making.

In some reinforcement learning tasks, the reward function is typically designed such that the agent receives a reward only when the output values satisfy the system requirements. Otherwise, the agent is continuously “punished”. This type of reward function is known as a sparse reward function. In simple environments like single-variable systems, using a sparse reward function can still yield good control results. However, in complex environments such as multivariable coupling systems, applying a sparse reward function can lead to difficulties in training and low training efficiency. Therefore, based on the characteristics of multivariable coupling systems, a dense reward function was designed in this study. For the loop $y_{1}$, the dense reward function is set as follows:

(8) $$r_{1}=\left\{\begin{array}{r} \alpha_{1}(-\frac{e_{1}^{2}}{\eta_{1}{}^{2}}+1),\;|e_{1}|\leq \eta_{1}\\ -\beta_{1}|e_{1}\left|,\;\;\;\;\;\;\;\;\;\;\;\right|e_{1}|>\eta_{1} \end{array}\right.$$

where $\alpha_{1}$ and $\beta_{1}$ are adjustable parameters, $\eta_{1}$ represents the threshold value of the error $e_{1}$ for loop 1. For different loops, with different system requirements for the error values, the value of η also varies. When c≠0, the characteristic of this reward function is that the agent can receive a non-zero reward regardless of the range of the error. Intuitively, this reward function can progressively provide rewards based on the performance of the agent. Even if the agent deviates significantly from the set value, it will still receive a non-zero reward. Therefore, the agent can also receive some motivation in the early stages of training. Similarly, for loop 2, the reward function is:

(9) $$r_{2}=\left\{\begin{array}{r} \alpha_{2}(-\frac{e_{2}^{2}}{\eta_{2}{}^{2}}+1),\;|e_{2}|\leq \eta_{2}\\ -\beta_{2}|e_{2}\left|,\;\;\;\;\;\;\;\;\;\;\;\right|e_{2}|>\eta_{2} \end{array}\right.$$

In summary, the reward function R designed for the multivariate coupled system in Figure 2 is shown in Equation (6). In this study, $\alpha_{1}$ equals 10, $\beta_{1}$ equals 0.5, $\eta_{1}$ equals 0.1, $\alpha_{2}$ equals 10, $\beta_{2}$ equals 1, and $\eta_{2}$ equals 0.1.

(10) $$R=r_{1}+r_{2}$$

### 3.5. Proximal Policy Optimization

In deep reinforcement learning, although simple and intuitive, policy-based methods can encounter training instability issues in practical applications. This instability often arises when the policy network is a deep model. During parameter updates using policy gradients, there is a risk of the policy significantly deteriorating due to overly large step sizes, thereby affecting training effectiveness. To address this problem, the trust region policy optimization (TRPO) algorithm [23] was introduced. TRPO aims to find a trusted region during updates, ensuring some level of safety in policy performance when updating within this region. While TRPO has been successful in many scenarios [24,25], its computational complexity results in a substantial computational load at each update step. As a response, the proximal policy optimization (PPO) algorithm [26] was introduced in 2017. PPO is based on the principles of TRPO but offers a more straightforward algorithmic implementation. PPO comes in two forms: PPO penalty and PPO clip. PPO penalty directly incorporates the constraint of KL divergence into the objective function using the Lagrange multiplier method, effectively turning it into an unconstrained optimization problem. During iterations, it continuously updates the coefficient in front of the KL divergence term. By adding this penalty term, PPO can restrict the magnitude of policy changes during each update, ensuring they stay within a reasonable range. This prevents introducing overly significant changes to the policy. On the other hand, PPO clip controls the size of policy changes by limiting the magnitude of policy updates. During each policy update, a truncation function clips the ratio of updated policy probabilities before and after the update. Typically, this truncation function restricts the updated probabilities to a fixed range, ensuring that the difference between new and old parameters does not become too large. These techniques in PPO help stabilize the training of deep reinforcement learning models, making them more suitable for practical applications.

The PPO algorithm contains two neural networks: the value network (critic) and the strategy network (actor). In this paper, the input layer of the critic network receives state information, the input dimension is the dimension of the state, the number of neurons in the hidden layer is 64 and 32, and the output layer contains one neuron, which is used for outputting the estimation of the state value. The actor network contains a public layer, an action mean estimation layer, and an action standard deviation estimation layer, in which the input layer of the public layer receives the state information. The number of neurons in the hidden layer is 64 and 32. Finally, the output layer has two neurons that are used to generate the parameters in the strategy, such as the mean and standard deviation of the action. These are then connected to the action mean estimation layer and the action standard deviation estimation layer, where the number of neurons in the hidden layer of the action mean estimation layer is 64 and 32. The number of neurons in the output layer is the dimensions in the space of actions. The number of neurons in the hidden layer of the action standard deviation estimation layer is 64 and 32. The number of neurons in the output layer is the dimension of the action space. Finally, SoftPlus is used as the activation function to ensure that the generated standard deviation is always positive.

Generally, a ReLU (Rectified Linear Unit) is often the default choice in reinforcement learning algorithms as the activation function in neural networks. However, experimental results [27] have shown that the PPO algorithm is better suited for using tanh as the activation function. Therefore, this study will verify whether there are certain advantages of using tanh as an activation function based on a multivariate coupled system. Additionally, in this study, the advantage function in the PPO algorithm was normalized using the mean and standard deviation of the current small-batch experiences. Furthermore, random initialization of setpoints within the system’s allowable range was performed at the beginning of each episode to enhance the algorithm’s adaptability to different setpoints for various loops in multivariable coupled systems. The algorithm of this study is shown in Algorithm 1:
**Algorithm 1:** Proximal policy optimization (PPO) for a multivariable systemInitialization: actor function parameters θ, critic function parameters ϕ, hyperparameters: truncation factor ε, number of sub-iterationsM,B for *k* = 0, 1, 2, … do Randomly initialize setpoints SP within the range allowed for each loop of a multivariable coupling system. Enforce policy $\pi_{\theta_{k}}$ in the environment, and save the track $D_{k}=\text{\{}\tau_{i}\text{\}}$ Calculated rewards $R_{t}$ Calculate the advantage function ${\overset{\wedge }{A}}_{t}$ based on the current critic function $V_{\phi_{k}}$ Normalizing the ${\overset{\wedge }{A}}_{t}$ using the mean and standard deviation of the current small batch experience for m∈{1,⋯,M} do  Computing importance sampling weights:   $l_{t}(\theta^{’})=\frac{\pi_{\theta }(A_{t}|S_{t})}{\pi_{\theta_{\mathrm{old}}}(A_{t}|S_{t})}$  Using the Adam stochastic gradient ascent algorithm to maximize the objective function of PPO clip to update the policy:   $\theta_{k+1}=\underset{\theta }{\arg\;\max}\frac{1}{|D_{k}|T}\sum_{\tau \in D_{k}}\sum_{t=0}^{T}\min(l_{t}(\theta^{’})A^{\pi_{\theta_{\mathrm{old}}}}(S_{t},A_{t}),\mathrm{clip}(l_{t}(\theta^{’}),1-\varepsilon ,1+\varepsilon )A^{\pi_{\theta_{\mathrm{old}}}}(S_{t},A_{t}))$ end for for b∈{1,⋯,B} do  The critic function is learned by minimizing the mean square error using the gradient descent algorithm:   $\phi_{k+1}=\underset{\phi }{\arg\;\min}\frac{1}{|D_{k}|T}\sum_{\tau \in D_{k}}\sum_{t=0}^{T}{\left(V_{\phi }(S_{t})-R_{t}\right)}^{2}$ end for end for

## 4. Experiment and Result Analysis

**Figure 3:**
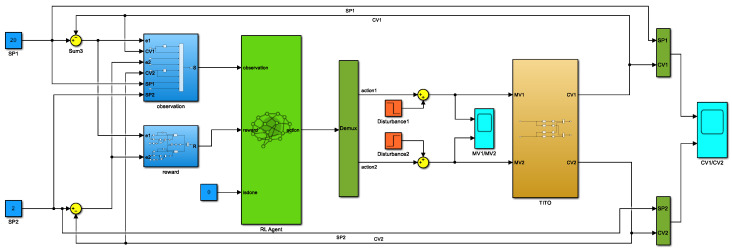
Control system based on deep reinforcement learning.

The TITO system in Section 2 was used as an environment to build the deep-reinforcement-learning-based control system structure in Simulink, as shown in Figure 3.

To better simulate the external interference signals, such as noise present in the natural environment, random interference signals were added to the input of the TITO system to strengthen the anti-interference capability of the proposed method in this study. The settings of the primary hyperparameters during the training process of the PPO algorithm are shown in Table 1.

Experimental validation has demonstrated that training the PPO algorithm with ReLU as the activation function in a multivariable coupled system environment is challenging. The average reward values during training can suddenly become very small, making it impossible to compare the average reward curves on the same coordinate axis. This may be because the ReLU activation function may lead to the problem of a vanishing gradient or gradient explosion in some cases, which will affect the stability of the optimization process. In contrast, in this study, tanh was chosen as the activation function because it maintains the output in the range [–1, 1], which helps to minimize the effect of the gradient problem and improves the algorithm’s stability. This stability is essential for controlling multivariate coupled systems, which often contain complex interactions and require a stable learning process for accurate control.

**Figure 4:**
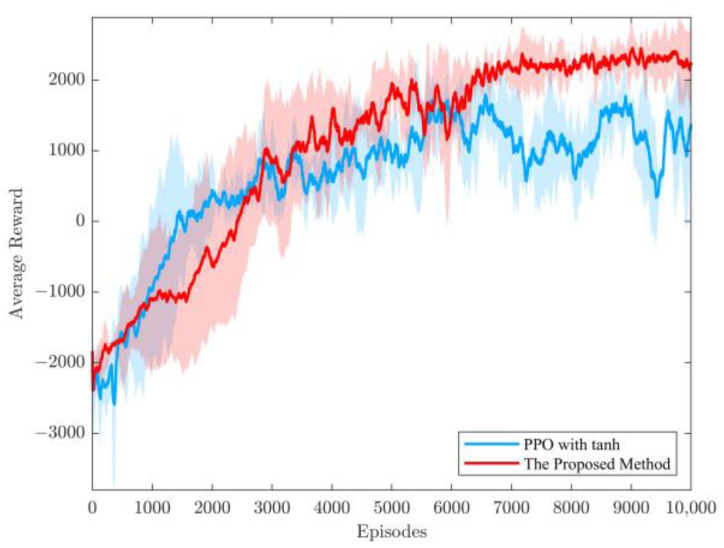
Average reward curve of the proposed method in this paper. Shaded areas indicate confidence intervals for each method.

The approach proposed in this paper, based on tanh as the activation function, includes normalizing the advantage function. The resulting average reward curve is depicted in Figure 4. To ensure fairness, the PPO algorithm employed the same neural network architecture, the same number of neurons, and the same learning rate. The experiments were conducted three times with different random seeds, each episode consisting of 10,000 rounds, and the recorded results represent the average reward values every 50 rounds. In order to ensure a thorough assessment, this study calculated the mean and standard deviation for each method and, in turn, the confidence intervals, where the mean reflects the central tendency, the standard deviation reflects the dispersion of the data, and the confidence intervals provide information on the confidence level of the estimate of the mean in order to estimate the reliability of the mean. This is important in assessing the statistical significance of the findings and the robustness of the conclusions of this study.

**Figure 5:**
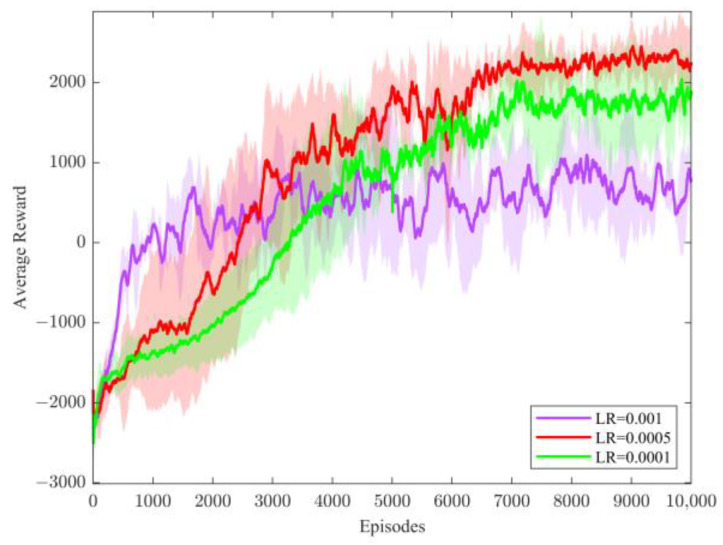
Average reward curves of the proposed method in this paper for different strategy learning rates. Shaded areas indicate confidence intervals for each method.

For the method proposed in this paper, to verify the effect of learning rate (LR) in the policy network on the performance of the algorithm, three different learning rates were selected for comparison, and the average reward curves are shown in Figure 5.

Figure 5 shows the average reward variation with episodes for the method proposed in this paper with different learning rates of the policy network selected. The method requires an appropriate learning rate to maintain the balance between learning and exploration, and the results show that when LR = 0.0001, the algorithm cannot perform sufficient exploration; when LR = 0.001, a more significant learning rate improves the exploration capability but negatively affects the convergence speed, resulting in an unstable learning process; however, when LR = 0.0005, the algorithm exhibits sufficient exploration capability and converges well. Therefore, LR = 0.0005 was chosen as the best performance of the algorithm.

To verify the control effectiveness of the proposed method in this paper, performance comparisons were made with decentralized control, decoupled control, and conventional PPO control. All computations were performed on a standard PC (Win11, AMD 4600H CPU@3.00 GHz, 16 GB) in MATLAB R2022b.

### 4.1. Control Performance Experiment

**Figure 6:**
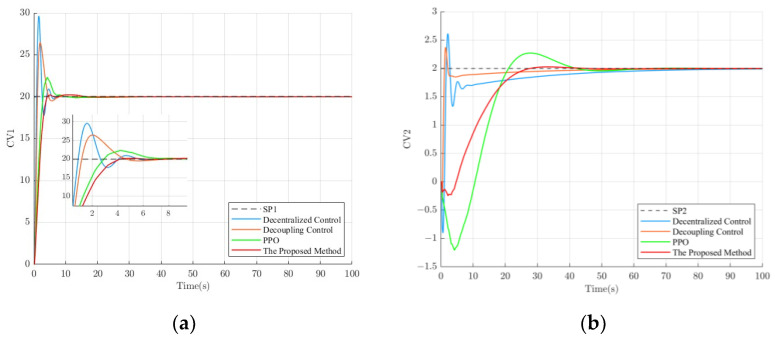
The control effect of different control methods. (**a**) The control effect of loop 1; (**b**) The control effect of loop 2.

To verify the control performance of the proposed method, the setpoints were set to 20 for SP1 and 2 for SP2, and the control effects of different methods are shown in Figure 6.

As is shown in Figure 6, the proposed method exhibits a relatively slower rise time compared to the other control methods. However, it outperforms the other methods in terms of settling time and overshoot. This may be attributed to the trade-offs made by the deep reinforcement learning agent during its trial-and-error learning process to achieve a balance between system stability, accuracy, and speed (maximizing long-term rewards). Among the control methods, decentralized control exhibits the highest overshoot. Although decoupling control improves the overshoot to some extent compared to decentralized control, it still lags behind the deep-reinforcement-learning-based control method. Moreover, the deep-reinforcement-learning-based control method eliminates the hassle associated with designing a decoupling controller. The performance parameters for loop 1 (Figure 6a) and loop 2 (Figure 6b) under different control methods are summarized in Table 2.

According to Table 2, it can be observed that, in terms of overshoot, compared to decentralized control, decoupling control, and PPO, the proposed method in loop 1 improved by 47.06%, 31.21%, and 10.54%, respectively, while in loop 2, it improved by 30.02%, 17.47%, and 12.25%, respectively. This is crucial for industrial processes that require high stability. In some aspects, the slower rise time of the method proposed in this paper is a trade-off, especially when the operator needs more time to adapt to the system state changes or when the system itself is more sensitive to sharp control actions. Meanwhile, this study profoundly analyzed the reasons behind the slower rise time and finally found that the design of the reward function has a significant impact on this result. Hence, it is suggested that, when facing industrial processes that require a fast rise time, a more appropriate reward function can be selected, for example, by considering adding the rise time metric to the reward function’s design. It is worth mentioning that the method proposed in this paper can take the lead in meeting the system requirements with a more stable trend using a shorter regulation time.

**Figure 7:**
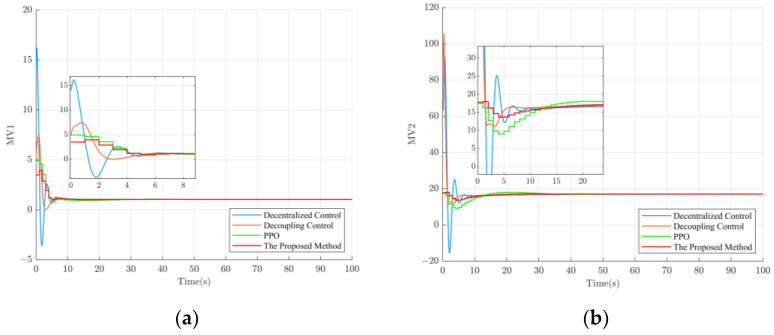
MV values of different control methods. (**a**) MV values for loop 1; (**b**) MV values for loop 2.

In addition, in industrial processes, the control variables are realized through actuators such as control valves or variable frequency pumps. The greater the variation range of the control variables, the higher the wear on equipment and instruments such as actuators. By reducing the variation range of the control variables, system stability and reliability can be improved, the service life of actuators and equipment can be prolonged, and maintenance costs can be reduced. Therefore, the magnitude of control variable variation is also a criterion for evaluating the performance of a controller. The variation in control variables for the proposed method and several other control methods is shown in Figure 7.

From Figure 7, it can be observed that the control performance in Figure 6 is closely related to the variation in the manipulated variable. Decentralized control and decoupling control exhibit large initial changes in the manipulated variable, resulting in significant overshoot. Moreover, due to the large adjustment range of the manipulated variable, it can be achieved in simulation experiments but is challenging to implement in real physical systems. In comparison to decentralized control and decoupling control, the deep reinforcement learning agent, after continuous trial and error, can better determine the range of control values required to reach the system setpoint, resulting in smaller overshoot.

### 4.2. Tracking and Interference Suppression Performance Experiment

**Figure 8:**
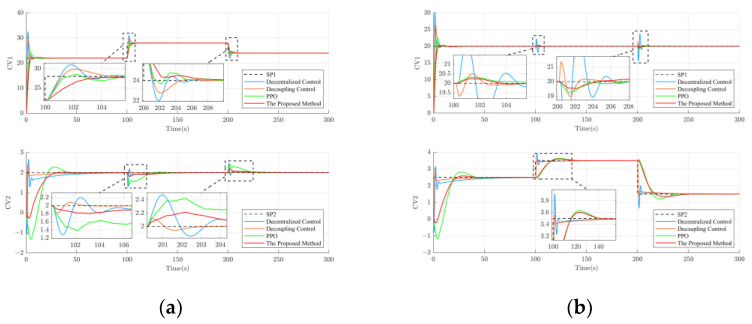
Results of tracking and disturbance rejection performance for different control methods. (**a**) Experimental results of the tracking performance for loop 1 and the disturbance rejection performance for loop 2; (**b**) Experimental results of the disturbance rejection performance for loop 1 and the tracking performance for loop 2.

To verify the tracking and disturbance rejection performance of the proposed method, separate 300 s simulation experiments were conducted for loop 1 and loop 2. The experimental setup was as follows: Firstly, the setpoint of loop 2 was kept constant, while the setpoint of loop 1 increased to 28 at t = 100 s and decreased to 24 at t = 300 s. Since the controlled object is a multivariable coupled system, the variation in the setpoint of loop 1 can be considered as a disturbance relative to loop 2. This setup allows the assessment of different control methods in terms of their ability to suppress disturbances in the system. The experimental results are shown in Figure 8a. Similarly, by keeping the setpoint of loop 1 constant and increasing the setpoint of loop 2 to 3.5 at t = 100 s and decreasing it to 1.5 at t = 300 s, the disturbance rejection performance of different control methods can be evaluated. The experimental results are shown in Figure 8b.

As is shown in Figure 8a, the proposed method exhibits a more stable tracking performance in loop 1. However, in loop 2, due to the coupling effect between the multivariable systems, the changes in loop 1′s setpoint cause significant fluctuations in loop 2. Therefore, decentralized control performs the worst in terms of disturbance rejection. On top of decentralized control, decoupling control, which incorporates decoupling controllers to eliminate interferences between different loops, achieves the best disturbance rejection performance. The proposed method ranks second in terms of disturbance rejection performance, closely following decoupling control. However, it is worth mentioning that when the setpoint of loop 2 changes, as shown in Figure 8b, the proposed method exhibits more stable disturbance rejection performance in loop 1 compared to decoupling control. Overall, it can be observed that the proposed method is generally more stable and reliable than other control methods.

### 4.3. External Noise Interference Suppression Performance Experiment

**Figure 9:**
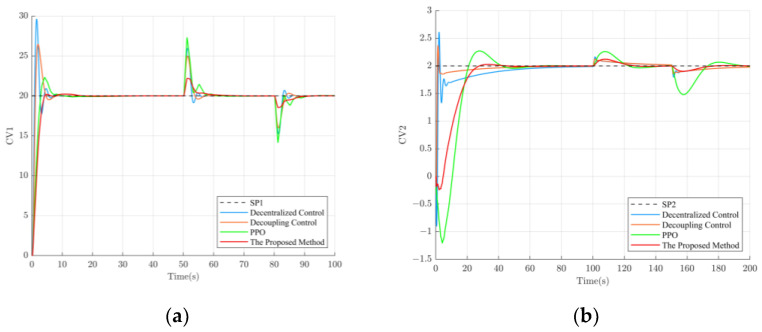
Results of external noise interference suppression performance for different control methods. (**a**) Experimental results of external noise interference suppression performance in loop 1; (**b**) Experimental results of external noise interference suppression performance in loop 2.

In order to verify the performance of the proposed method in the presence of external noise interference and to gain a more comprehensive understanding of its robustness, simulation experiments were conducted for 100 s and 200 s for loop 1 and loop 2, respectively. The external noise interference signals with amplitudes of 5 and −4 were applied to loop 1 at 50 s and 80 s, respectively, and the external noise interference signals with amplitudes of 4 and −5 were applied to loop 2 at 100 s and 150 s, respectively. The experimental results are shown in Figure 9.

As is shown in Figure 9a, in loop 1, the proposed method has the best performance among the methods explored in this paper due to the addition of a random disturbance signal with an amplitude between [−5, 5], which is capable of simulating external noise, during the training process. In loop 2, as is shown in Figure 9b, the performance of the method proposed in this paper is equal to that of decentralized and decoupled control. It is significantly better than the traditional PPO control, suggesting that adding external noise disturbance signals can improve the algorithm’s performance during training.

## 5. Conclusions

This paper introduces a deep-reinforcement-learning-based control method for multivariable coupled systems to address issues such as imprecise and unstable control resulting from the coupling between variables. Firstly, the structure and model of multivariable systems was analyzed and an example of the coupling characteristics between these systems was provided. Subsequently, considering the characteristics of multivariable coupled systems, reward functions and control structures were designed. A deep reinforcement learning agent was constructed using the PPO algorithm, utilizing tanh as the activation function and normalizing the advantage function. The agent selects actions for each loop based on the system’s state features, gradually approaching the optimal policy through updates based on different rewards obtained during interactions with the environment. Furthermore, to account for real-world noise and enhance the algorithm’s adaptability to different setpoints, random disturbance signals are introduced at the input of the controlled object during training, and setpoints for each loop are randomly initialized at the beginning of each episode. Simulation results demonstrate that the proposed approach achieves superior control performance and holds significant potential for multivariable coupled systems.

However, the method proposed in this paper is not only applicable to this particular system but also can be considered as applicable to other multivariable systems. The detailed descriptions of the state space representation, reward function design, neural network parameters, and algorithm hyperparameters provided in this paper can provide a reference basis for other researchers to apply the proposed method in other multivariable systems.

In the future, we will consider studying real scenarios to strengthen the credibility of the method proposed in this paper and explore other advanced deep reinforcement learning techniques or approaches.

## Figures and Tables

**Table 1:** PPO algorithm hyperparameter settings.

Hyperparameter	Setting
Sampling time	0.1 s
Simulation time	20 s
Number of trainings	10,000
Gamma	0.99
Number of critic network neurons	64, 32
Number of common layer neurons in the actor network	64, 32, 2
Number of mean layer neurons in the actor network	64, 32
Number of standard deviation layer neurons in the actor network	64, 32
Critic learning rate	0.001
Actor learning rate	0.0005
Mini batch size	256
Policy entropy	0.01

**Table 2:** Performance parameters of different control methods.

Loops	Control Methods	Rise Time (s)	Transient Time (s)	Overshoot (%)
Loop1	Decentralized Control	0.52	5.28	48.21
	Decoupling Control	0.65	6.26	32.36
	PPO	1.99	6.53	11.69
	The Proposed Method	2.60	3.91	1.15
Loop2	Decentralized Control	0.45	50.26	31.36
	Decoupling Control	0.44	30.64	18.81
	PPO	7.83	38.44	13.59
	The Proposed Method	14.86	25.08	1.34

## Data Availability

Not applicable.

## References

[1] Almeida A.M.D., Lenzi M.K., Lenzi E.K. (2020). A Survey of Fractional Order Calculus Applications of Multiple-Input, Multiple-Output (MIMO) Process Control. Fractal Fract..

[2] Mahapatro S.R., Subudhi B. (2022). A Robust Stability Region-Based Decentralized PI Controller for a Multivariable Liquid Level System. IEEE Syst. J..

[3] Liu J., Li P. (2021). Control and Real-Time Data Acquisition of an Experimental Platform for Stored Grain Aeration Study. Sensors.

[4] Zhang Y., Chai T., Wang D., Chen X. (2018). Virtual Unmodeled Dynamics Modeling for Nonlinear Multivariable Adaptive Control with Decoupling Design. IEEE Trans. Syst. Man Cybern. Syst..

[5] Abdul-Adheem W.R., Ibraheem I.K. (2019). Decoupled control scheme for output tracking of a general industrial nonlinear MIMO system using improved active disturbance rejection scheme. Alex. Eng. J..

[6] Rathnayake D.B., Bahrani B. (2023). Multivariable Control Design for Grid-Forming Inverters with Decoupled Active and Reactive Power Loops. IEEE Trans. Power Electron..

[7] Karimi A., Kammer C. (2017). A data-driven approach to robust control of multivariable systems by convex optimization. Automatica.

[8] Yousfi M., Ben Njima C., Garna T. (2022). Robust multimodel control for uncertain nonlinear MIMO systems based on ARX-Laguerre multimodel and LSDP approach. Int. J. Control..

[9] Belmonte L.M., Morales R., Fernández-Caballero A., Somolinos J.A. (2016). Robust Decentralized Nonlinear Control for a Twin Rotor MIMO System. Sensors.

[10] Xue C., Ding L., Wu X., Li Y., Song W. (2022). Model Predictive Control for Grid-Connected Current-Source Converter with Enhanced Robustness and Grid-Current Feedback Only. IEEE J. Emerg. Sel. Top. Power Electron..

[11] Zhong Z., del Rio-Chanona E.A., Petsagkourakis P. (2023). Tube-based distributionally robust model predictive control for nonlinear process systems via linearization. Comput. Chem. Eng..

[12] Cheng Y., Sun M.W., Sun Q. (2017). Multivariable Inverted Decoupling Active Disturbance Rejection Control and Its Application to a Distillation Column Process. Zidonghua Xuebao/Acta Autom. Sin..

[13] Wu Z., Liu Y., Li D., Chen Y. (2023). Multivariable active disturbance rejection control for compression liquid chiller system. Energy.

[14] Hajare V.D., Patre B.M., Khandekar A.A., Malwatkar G.M. (2017). Decentralized PID controller design for TITO processes with experimental validation. Int. J. Dyn. Control..

[15] Zhou B., Xu Y. (2007). Robust control of a 3-DOF hybrid robot manipulator. Int. J. Adv. Manuf. Technol..

[16] Schwenzer M., Ay M., Bergs T., Abel D. (2021). Review on model predictive control: An engineering perspective. Int. J. Adv. Manuf. Technol..

[17] Mnih V., Kavukcuoglu K., Silver D., Rusu A.A., Veness J., Bellemare M.G., Graves A., Riedmiller M., Fidjeland A.K., Ostrovski G. (2015). Human-level control through deep reinforcement learning. Nature.

[18] Yang Q., Cao W., Meng W., Si J. (2022). Reinforcement-Learning-Based Tracking Control of Waste Water Treatment Process Under Realistic System Conditions and Control Performance Requirements. IEEE Trans. Syst. Man Cybern. Syst..

[19] Shuprajhaa T., Sujit S.K., Srinivasan K. (2022). Reinforcement learning based adaptive PID controller design for control of linear/nonlinear unstable processes. Appl. Soft Comput..

[20] Zhu Y., Pan M., Zhou W., Huang J. (2022). Intelligent direct thrust control for multivariable turbofan engine based on reinforcement and deep learning methods. Aerosp. Sci. Technol..

[21] Zheng Y., Ji G. (2012). Approach of inverted decoupling suitable for high order multivariable system. J. Beijing Univ. Technol..

[22] Arulkumaran K., Deisenroth M.P., Brundage M., Bharath A.A. (2017). Deep Reinforcement Learning A brief survey. IEEE Signal Process. Mag..

[23] Schulman J., Levine S., Moritz P., Jordan M., Abbeel P. (2015). Trust Region Policy Optimization. Proceedings of the 32nd International Conference on International Conference on Machine Learning.

[24] Nachum O., Norouzi M., Xu K., Schuurmans D. (2017). Trust-PCL: An Off-Policy Trust Region Method for Continuous Control. arXiv.

[25] Weisenthal S., Thurston S., Ertefaie A. (2022). Relative Sparsity for Medical Decision Problems. Stat. Med..

[26] Schulman J., Wolski F., Dhariwal P., Radford A., Klimov O.J.A. (2017). Proximal Policy Optimization Algorithms. arXiv.

[27] Engstrom L., Ilyas A., Santurkar S., Tsipras D., Janoos F., Rudolph L., Madry A.J. (2020). Implementation matters in deep policy gradients: A case study on ppo and trpo. arXiv.

